# Supplementary material for: Single-neuron spiking variability in hippocampus dynamically tracks sensory content during memory formation in humans
Source: Nat Commun. 2025 Jan 2;16:236. doi: 10.1038/s41467-024-55406-4 (PMC11696175; doi:10.1038/s41467-024-55406-4)
Supplement: Supplementary file 1 — Supplementary Information [file 41467_2024_55406_MOESM1_ESM.pdf]

Supplemental figures

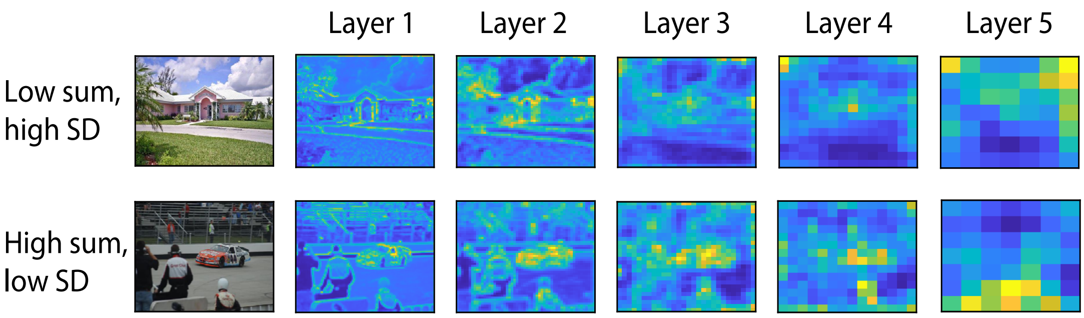

Figure S1. **Layer-wise average activation maps of two example images illustrating differences in feature metrics**. Both rows show average activation maps from all VGG16 max pooling layers (corresponding to layers 3, 6, 10, 14, & 18). The upper row contains maps of an image that comes with a relatively low spatial sum (across pixels per layer and feature) but a high SD, tracing back to the uneven distribution of salient spots across space (grass vs. house). The lower row contains maps of an image with relatively high sum but low SD (salient spots distributed relatively evenly). Example images used here from [20] (https://creativecommons.org/licenses/by/4.0/).

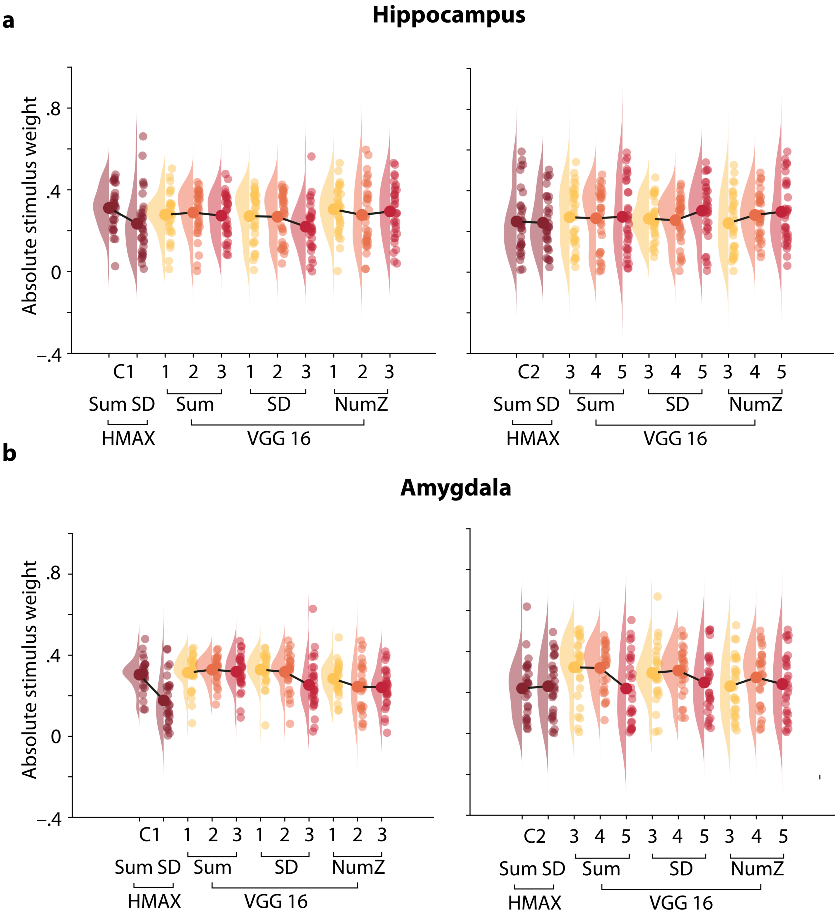

Figure S2. Participant-, layer- and model wise patterns of absolute stimulus weights. Absolute weights capture the relative importance of stimulus features for the observed latent correlation. (**a**) absolute weights for early (left) and late (right) layer models, grouped for Computational vision models (HMAX vs VGG16) and feature metrics (Sum, SD, number of non-zero entries NumZ). Dots represent absolute weights from individual PLS models, large non-transparent dots capture grand averages and are connected by black lines (n = 34). (**b**) As in panel a, but for models based on amygdala neurons. Here, later layers seem to be given lower weights as compared to earlier layers.

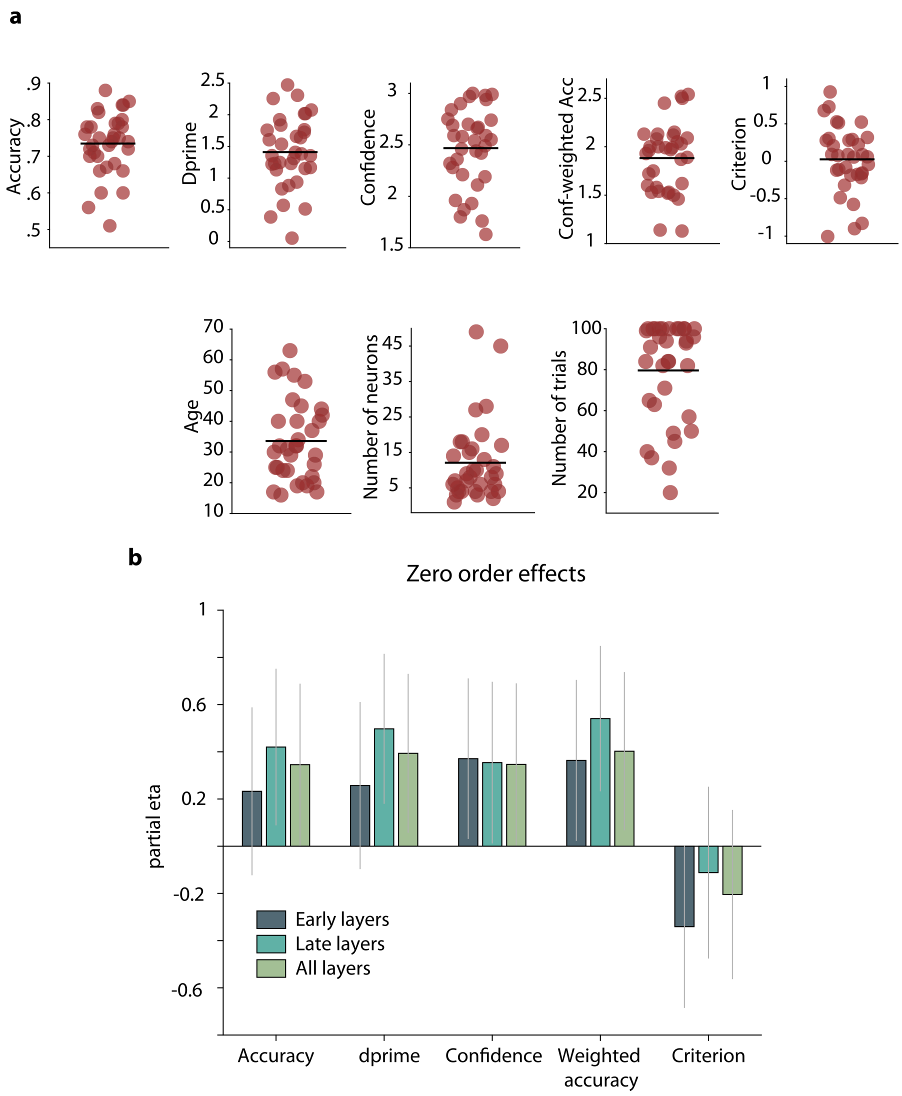

Figure S3. Descriptive plots of inter-individual variables and their link to spike-image coupling. (a) Scatter plots of all relevant behavioural variables, displaying across-participant averages (horizontal black line; each dot represents one participant, n = 34). (b) Zero-order relationships between individual hippocampal spike PE to image feature coupling and behavioural metrics. Vertical lines depict bootstrapped 95% confidence intervals. Memory accuracy and dprime are positively linked to all coupling estimates, but only late and all layer coupling estimates are linked significantly. Confidence is linked positively to coupling estimates from early, late, and all layer models. Similarly, confidence-weighted accuracy is positively correlated with all layer-wise coupling estimates, strongest for coupling to late layers. Response criterion is not significantly linked to any of the three coupling metrics.

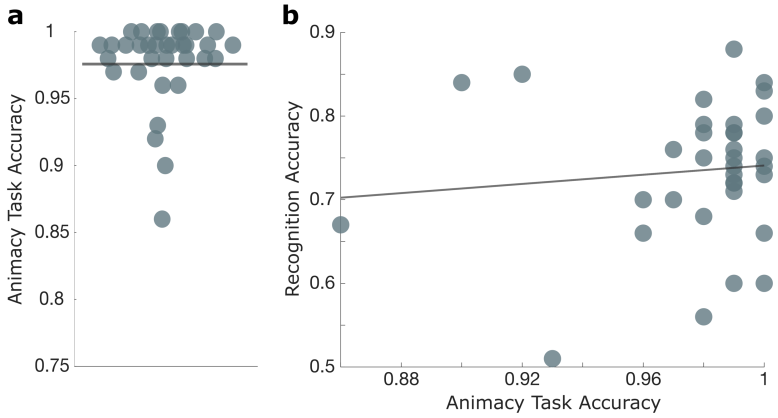

Figure S4: Control analysis of the animacy task. (a) Accuracy in the animacy task across subjects. **(b)** Correlation between memory recognition and animacy task accuracy (r=.1, *p*=.56; n=34).
