# Supplementary material for: Single-neuron spiking variability in hippocampus dynamically tracks sensory content during memory formation in humans
Source: Nat Commun. 2025 Jan 2;16:236. doi: 10.1038/s41467-024-55406-4 (PMC11696175; doi:10.1038/s41467-024-55406-4)
Supplement: Supplementary file 2 — Reporting Summary [file 41467_2024_55406_MOESM2_ESM.pdf]

nature portfolio

Corresponding author(s): Douglas D. Garrett

Last updated by author(s): Sep 19, 2024

Reporting Summary

Nature Portfolio wishes to improve the reproducibility of the work that we publish. This form provides structure for consistency and transparency in reporting. For further information on Nature Portfolio policies, see our Editorial Policies and the Editorial Policy Checklist.

Statistics

For all statistical analyses, confirm that the following items are present in the figure legend, table legend, main text, or Methods section.

n/a	Confirmed	
☐	☒	The exact sample size (*n*) for each experimental group/condition, given as a discrete number and unit of measurement
☐	☒	A statement on whether measurements were taken from distinct samples or whether the same sample was measured repeatedly
☐	☒	The statistical test(s) used AND whether they are one- or two-sided<br>*Only common tests should be described solely by name; describe more complex techniques in the Methods section.*
☐	☒	A description of all covariates tested
☐	☒	A description of any assumptions or corrections, such as tests of normality and adjustment for multiple comparisons
☐	☒	A full description of the statistical parameters including central tendency (e.g. means) or other basic estimates (e.g. regression coefficient) AND variation (e.g. standard deviation) or associated estimates of uncertainty (e.g. confidence intervals)
☐	☒	For null hypothesis testing, the test statistic (e.g. *F*, *t*, *r*) with confidence intervals, effect sizes, degrees of freedom and *P* value noted<br>*Give P values as exact values whenever suitable.*
☒	☐	For Bayesian analysis, information on the choice of priors and Markov chain Monte Carlo settings
☐	☒	For hierarchical and complex designs, identification of the appropriate level for tests and full reporting of outcomes
☐	☒	Estimates of effect sizes (e.g. Cohen's *d*, Pearson's *r*), indicating how they were calculated

Our web collection on statistics for biologists contains articles on many of the points above.

Software and code

Policy information about availability of computer code

Data collection	See open data availability statement in the section below.
Data analysis	A full account of all data processing and analyses can be found in our repo at: https://github.com/LNDG/SpikeVar

For manuscripts utilizing custom algorithms or software that are central to the research but not yet described in published literature, software must be made available to editors and reviewers. We strongly encourage code deposition in a community repository (e.g. GitHub). See the Nature Portfolio guidelines for submitting code & software for further information.

Data

Policy information about availability of data

All manuscripts must include a data availability statement. This statement should provide the following information, where applicable:

- Accession codes, unique identifiers, or web links for publicly available datasets
- A description of any restrictions on data availability
- For clinical datasets or third party data, please ensure that the statement adheres to our policy

We re-analyzed human hippocampus and amygdala single neuron activity from a previously published dataset of 42 patients (total number of sessions = 65) undergoing surgery for intractable epilepsy who performed an encoding and recognition memory task. A full account of this dataset is available in: Faraut, M.C.M. et al. Scientific Data 5, 180010 (2018). https://www.nature.com/articles/sdata201810. https://datadryad.org/stash/dataset/doi:10.5061/dryad.46st5

Research involving human participants, their data, or biological material

Policy information about studies with human participants or human data. See also policy information about sex, gender (identity/presentation), and sexual orientation and race, ethnicity and racism.

Reporting on sex and gender	42 subjects (15 female)
Reporting on race, ethnicity, or other socially relevant groupings	n/a
Population characteristics	42 patients (total number of sessions = 65) undergoing surgery for intractable epilepsy; Age (16-70 years);
Recruitment	see https://www.nature.com/articles/sdata201810
Ethics oversight	Protocols were approved by the institutional review boards of the Cedars-Sinai Medical Center, Huntington Memorial Hospital and the California Institute of Technology.

Note that full information on the approval of the study protocol must also be provided in the manuscript.

Field-specific reporting

Please select the one below that is the best fit for your research. If you are not sure, read the appropriate sections before making your selection.

☒ Life sciences ☐ Behavioural & social sciences ☐ Ecological, evolutionary & environmental sciences

For a reference copy of the document with all sections, see nature.com/documents/nr-reporting-summary-flat.pdf

Life sciences study design

All studies must disclose on these points even when the disclosure is negative.

Sample size	We re-analyzed human hippocampus and amygdala single neuron activity from a previously published dataset of 42 patients (total number of sessions = 65) undergoing surgery for intractable epilepsy who performed an encoding and recognition memory task.
Data exclusions	While further details on task, recordings, and basic performance can be found elsewhere20,52, it is important to note that we limited our analyses of neural activity to the first encoding session of n = 34 patients whose recordings included active hippocampus neurons (average neuron PE > .0001; 12±11 hippocampal neurons per individual and session, total N = 411).
Replication	All analyses have been verified by two scientists to be reproducible via the code base in our study repo: https://github.com/LNDG/SpikeVar
Randomization	There are no experimental groups in the current study.
Blinding	Blinding was not relevant in the current design. All subjects consented to take part in the current encoding and recognition task.

Reporting for specific materials, systems and methods

We require information from authors about some types of materials, experimental systems and methods used in many studies. Here, indicate whether each material, system or method listed is relevant to your study. If you are not sure if a list item applies to your research, read the appropriate section before selecting a response.

Materials & experimental systems

n/a	Involved in the study	
☒	☐	Antibodies
☒	☐	Eukaryotic cell lines
☒	☐	Palaeontology and archaeology
☒	☐	Animals and other organisms
☒	☐	Clinical data
☒	☐	Dual use research of concern
☒	☐	Plants

Methods

n/a	Involved in the study	
☒	☐	ChIP-seq
☒	☐	Flow cytometry
☒	☐	MRI-based neuroimaging

Plants

Seed stocks	n/a
Novel plant genotypes	n/a
Authentication	n/a
